# The Teacher–Assistant–Student Collaborative and Competitive Network for Brain Tumor Segmentation with Missing Modalities

**DOI:** 10.3390/diagnostics15121552

**Published:** 2025-06-18

**Authors:** Junjie Wang, Huanlan Kang, Tao Liu

**Affiliations:** School of Mathematics and Statistics, Northeastern University at Qinhuangdao, Qinhuangdao 066004, China; 202215056@stu.neuq.edu.cn (J.W.); 202211393@stu.neuq.edu.cn (H.K.)

**Keywords:** brain tumor segmentation, missing modalities, knowledge distillation, medical image segmentation, deep learning, collaboration and competition

## Abstract

**Background**: Magnetic Resonance Imaging (MRI) provides rich tumor information through different imaging modalities (T1, T1ce, T2, and FLAIR). Each modality offers distinct contrast and tissue characteristics, which help in the more comprehensive identification and analysis of tumor lesions. However, in clinical practice, only a single modality of medical imaging is available due to various factors such as imaging equipment. The performance of existing methods is significantly hindered when handling incomplete modality data. **Methods**: A Teacher–Assistant–Student Collaborative and Competitive Net (TASCCNet) is proposed, which is based on traditional knowledge distillation techniques. First, a Multihead Mixture of Experts (MHMoE) module is developed with multiple experts and multiple gated networks to enhance information from fused modalities. Second, a competitive function is formulated to promote collaboration and competition between the student network and the teacher network. Additionally, we introduce an assistant module inspired by human visual mechanisms to provide supplementary structural knowledge, which enriches the information available to the student and facilitates a dynamic teacher–assistant collaboration. **Results**: The proposed model (TASCCNet) is evaluated on the BraTS 2018 and BraTS 2021 datasets and demonstrates robust performance even when only a single modality is available. **Conclusions**: TASCCNet successfully addresses the challenge of incomplete modality data in brain tumor segmentation by leveraging collaborative knowledge distillation and competitive learning mechanisms.

## 1. Introduction

With the rapid advancement of medical imaging technology, magnetic resonance imaging (MRI) has become a crucial tool in brain tumor diagnosis [1,2,3,4,5,6,7]. It plays a significant role in detecting and segmenting gliomas. In multimodal brain tumor segmentation, four common MRI modalities are typically used: T1-weighted (T1), enhanced T1-weighted (T1ce), T2-weighted (T2), and fluid-attenuated inversion recovery (FLAIR) [1,2,3]. T1 and T1ce mainly evaluate the tumor core, while T2 and FLAIR assess surrounding edema [4,5]. However, factors such as imaging conditions, scanning equipment, or data corruption often result in missing modalities in clinical practice [6,7]. Consequently, the performance of existing methods decreases significantly when handling incomplete modal data. As a result, a key research question has emerged: how can limited modal information be effectively utilized to approximate or match the segmentation performance achieved with complete modal data? To address this challenge, two mainstream approaches have been proposed in the academic community for handling missing modalities.

**Figure 1:**
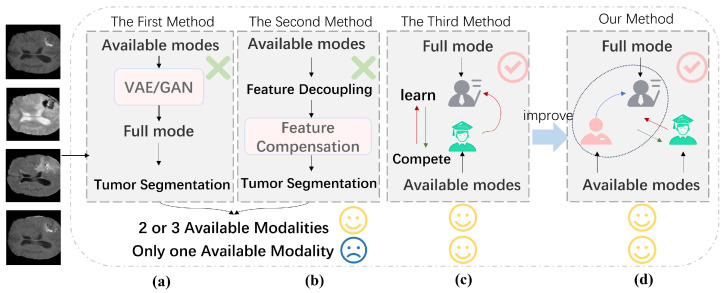
Description and improvement of three methods. (**a**,**b**) demonstrate resolution of missing modality by modality generation or modality fusion. (**c**) is the traditional knowledge distillation. (**d**) is the method proposed in this study.

As shown in Figure 1, the first method generates the missing modality from the available modalities [8,9]. The second method extracts features from the available modalities, maps them into a learnable latent space, and combines them using fusion techniques [10,11,12,13,14]. However, due to the limited features of a single modality, unsatisfactory results are often produced by these two methods. The above two methods aim to transfer information from the accessible modalities to the missing modality. This concept of knowledge transfer inspires us to apply knowledge distillation methods to address the brain tumor segmentation problem with a single modality.

The traditional knowledge distillation network [15] structure is composed of three parts: the teacher network, the student network, and the knowledge transfer network. However, due to deficiencies in the network structure, the traditional knowledge distillation methods have been found to exhibit certain limitations when applied to the brain tumor segmentation task in a unimodal setting. Therefore, it is necessary to analyze and improve the three components of the knowledge distillation network.

First, in the processing of multimodal feature inputs, traditional teacher networks typically focus on improving the fusion of multimodal features. Zhang et al. [16] proposed the Multimodal Medical Transformer (mmformer) for incomplete multimodal learning in brain tumor segmentation, which utilizes an interactive attention mechanism. Similarly, Liu et al. [17] introduced the Multimodal Representation Learning for Brain Tumor Segmentation with Missing Modalities (M3AE), which employs Masked Autoencoders (MAEs) modules. However, for the teacher network, the processing of multimodal feature inputs by the teacher model can be further enhanced.

Second, the student network also needs to improve its knowledge learning approach to enhance segmentation performance. Traditional knowledge transfer methods are singular in nature. Wang et al. [18] proposed Prototype Knowledge Distillation (ProtoKD), and Hu et al. [19] introduced Knowledge Distillation Net (KD-Net), both of which simply align the hidden layer features of the student network with those of the teacher network. These traditional student networks simply imitate the teacher networks, which limits their performance potential to that of the teacher networks.

Third, traditional knowledge transfer networks involve distilled knowledge being received by the student network from the teacher network after its parameters are frozen. According to the Dual Disentanglement Network (D2-Net) by Yang et al. [20], the teacher’s parameters are typically frozen during knowledge transfer, lacking a self-updating mechanism to further enhance its performance. It is impossible to update the parameters of the teacher network according to the input sample of the student network to better transfer knowledge, which prevents the further improvement of the overall performance of the network. In real-world scenarios, new knowledge is acquired by teachers while being imparted to students, indicating the need for a model that allows the teacher to self-update during the teaching process.

Based on the above background, this study proposes a Teacher–Assistant–Student Collaborative and Competitive Network (TASCCNet). The goal of TASCCNet is to improve segmentation performance when only a single modality is available. To enhance the ability to amplify multimodal features, a teacher network based on the Multihead Mixture of Experts (MHMoe) framework is proposed. The input channels are divided into multiple heads by the MHMoE, and multiple experts and gating mechanisms are employed to handle modality information across different channels. To enhance the learning capability of the student model, inspiration is drawn from Generative Adversarial Networks (GANs) [21] to create a competitive function, which encourages the student network to compete with the teacher network while learning. Our student network is designed to accept only a single MRI modality as input and, through knowledge distillation and competitive learning mechanisms, it strives to achieve or approach the segmentation performance of the teacher network trained with complete multimodal data. In addition, the auxiliary network that is inspired by the human visual mechanism is proposed [22]. It can absorb new knowledge and make up for the content and structural information lacking in the teacher’s network.

The main contributions of this work are as follows:

- A Multihead Mixture of Experts (MHMoE) mechanism was introduced to enhance the model’s feature representation.
- The competitive function was effectively integrated into the model, improving the student network’s performance.
- An auxiliary network inspired by the human visual system was proposed to address the limitations of knowledge updating in the teacher network.
- Experimental results demonstrated that both the teacher model and the student network performed well, with the student network surpassing the teacher network in some cases.

The structure of the following sections are as follows: Section 2 provides a detailed introduction to the TASCCNet framework. Section 3 presents the testing of the proposed model on BraTS2018 and BraTS2021 datasets, followed by qualitative and quantitative analyses. Section 4 summarizes the contributions of this work and offers insights into future research directions.

## 2. Materials and Methods

**Figure 2:**
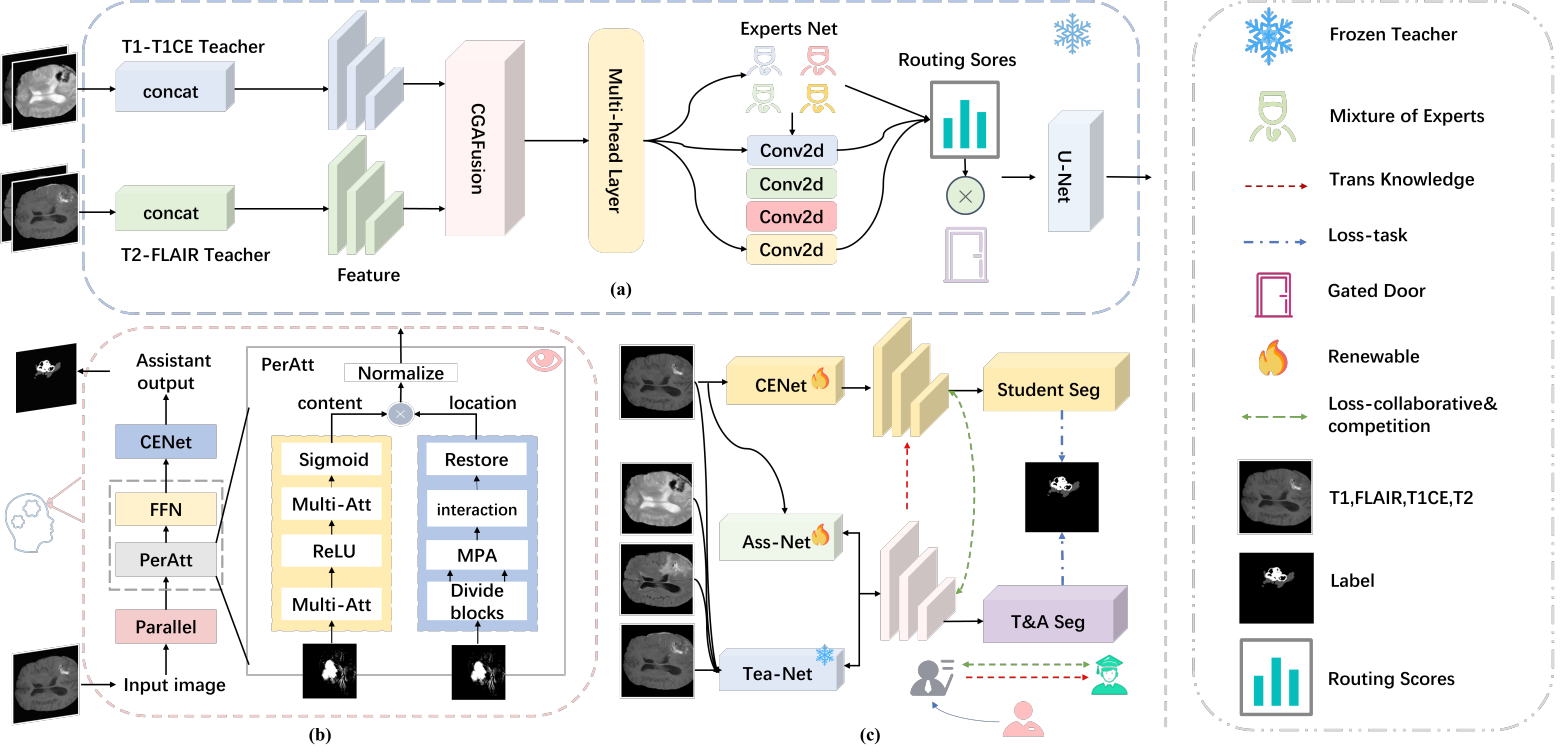
The complete flow chart of this study. (**a**) shows the CGAMHMoE-UNet teacher model, with T1-T1CE and T2-FLAIR inputs. Features are fused by CGAFUSION, enhanced by MHMOE, and segmented by UNet. (**b**) shows that the assistant model consists of two parallel branches, MHA and MPA, with CENet as the encoding and solver for the helper model segmentation. (**c**) shows the network architecture. The parameters of the teacher model are frozen after training. The segmentation results of teacher model and assistant model are weighted and added to obtain the output of teacher–assistant network. The student model is segmented by CENet code and solver. In the process of knowledge distillation, the student model and the teacher–assistant network model both learn and compete to obtain the final segmentation results.

The TASCCNet framework consists of three modules: the teacher module, the assistant module, and the student module. First, a teacher network is designed based on U-Net, which accepts full modality input and enhances multimodal features through the MHMoE module. In addition, an assistant module is introduced, which is inspired by the human eye’s peripheral vision mechanism. The assistant model accepts the same unimodal input as the student model. By combining the outputs of the assistant and teacher models, the teacher–assistant network produces an output that allows for self-updating while keeping the teacher model’s parameters frozen. Finally, a competition function is introduced. During training, the student model learns from the teacher–assistant network through a collaborative mechanism, while optimizing itself through a competitive mechanism. The overall framework is shown in Figure 2.

### 2.1. CGAMHMoE-UNet Teacher Model

**Figure 3:**
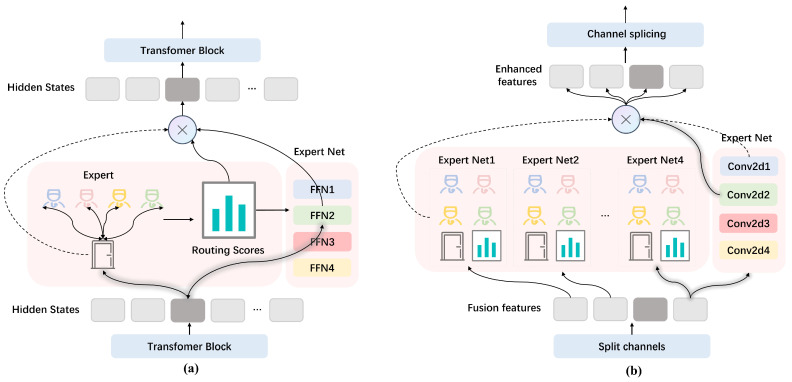
The traditional MOE model and the MHMOE model of this study. (**a**) is the traditional MOE model, and (**b**) is the MHMOE model of this study.

Each modality (T1, FLAIR, T1CE, T2, etc.) provides different tissue contrasts and lesion characteristics. While the information across modalities shares some commonalities, it also presents unique differences. Simply fusing all features in the same module may prevent the model from fully utilizing the distinctiveness of each modality. Inspired by MoE [23], this study proposes the new MHMoE model. As shown in Figure 3, unlike the traditional MoE [24] which uses a single gated network, the MHMoE model employs multiple gated networks with multiple experts.

The core idea of the MHMoE is to divide the image space into multiple channels. Each channel has several experts, and a corresponding gated network dynamically weights the outputs of these experts. Each expert focuses on a specific subset of the problem space. This “specialization” approach allows the model to learn a more diverse set of features and patterns with greater accuracy, reducing the risk of overfitting a single model. The gated networks enable the model to dynamically select the expert best suited for the current input, offering greater flexibility in handling different types of data.

Additionally, the expert network in the MHMoE consists of a series of convolutional layers. The traditional MoE typically uses feedforward layers to process one-dimensional or flattened input data, but this flattening operation disrupts the spatial structure of the image. As a result, critical spatial information is lost, reducing the model’s ability to recognize brain structures. Feedforward layers also primarily rely on global features for decision making, which may lead to the neglect of local features. In medical images, lesion areas are often concentrated in small, localized regions. Ignoring these local features can impair the model’s ability to detect subtle lesions. The MHMoE addresses this by using convolutional layers that retain the spatial structure of the input feature maps to extract local features. The design, by sharing parameters across different locations with the same convolutional kernel, aims to reduce the number of parameters and manage computational load.

MHMoE employs a multihead gating mechanism, allowing for the processing of multimodal features at a finer granularity. This approach maximizes the use of experts and enhances knowledge sharing and integration across expert networks. Specifically, the MHMoE divides the input channel features into multiple subsets (heads), with each head assigned its own independent gating network. Each gating network selects experts specifically for the feature subset it processes. As a result, multiple heads can simultaneously select different experts, significantly increasing the number of activated experts and improving the model’s ability to learn complex features. Moreover, by splitting input features into multiple heads and processing them in parallel with several experts, the model captures finer details of different modal features across heads, leading to better integration and utilization of multimodal data.

Let the input fusion feature be X with dimensions B×C×H×W, where B is the batch size, C is the number of channels, and H and W are the height and width of the feature map, respectively. In the MHMOE module, we divide the input channel features into four heads. For each head $X_{i}$, we configure k=4 expert subnetworks $E_{j}$. All expert subnetworks share the same architecture, which consists of two consecutive 3×3 convolutional layers and one ReLU activation function, each of which $E_{j}$ processes to generate the expert output $E_{j}\left(X_{i}\right)$.

(1) $$E_{j}\left(X_{i}\right)=Conv_{j}\left(X_{i}\right).$$

Each head $X_{i}$ generates activation weights $G_{\mathrm{ij}}$ through an independent gating network $G_{i}$ that determines which experts are activated:

(2) $$G_{i}=softmax\left(Gate\left(X_{i}\right)\right),$$

where the dimension of $G_{i}$ is B×k×H×W, denoting the weight for each expert j. For each head $X_{i}$, the outputs of all experts are fused as

(3) $$O_{i}=\sum_{j=1}^{k}G_{i,j}\cdot E_{j}\left(X_{i}\right).$$

Finally, the outputs of all the heads are spliced in the channel dimension to form the final augmented feature output O:

(4) $$O=Concat(O_{1},O_{2},\ldots ,O_{n}).$$

This fine-grained expert activation per head is particularly beneficial when dealing with fused multimodal inputs, as different heads can learn to focus on and process information pertinent to specific modalities or their combined characteristics more effectively. The teacher model receives full modality inputs, with T1 and T1CE as well as FLAIR and T2 being complementary. In this paper, T1 and T1CE are paired, and FLAIR and T2 are paired; T1 and T1CE are beneficial for evaluating the tumor core, while T2 and FLAIR are beneficial for assessing peritumoral edema. This pairing enables the teacher network to better exploit synergistic information between different modalities during training. And the inputs are concatenated by channels before being passed to the teacher network.

The CFAFusion module from Detail Enhancement Convolution and Content Guided Attention Net (DEA-Net) [25] is used for feature fusion (CGAFusion in Figure 2). The channel weights $W_{c}$ and spatial weights $W_{s}$ are computed, respectively, where $W_{c}$ compresses the input features in the channel dimension using global average pooling and then goes through two 1×1 convolutional layers with ReLU activation function in the middle, and $W_{s}$ compresses the input features in the spatial dimension using global average pooling and global maximum pooling and is processed through 7×7 convolutional layers, which ultimately connects the two channels. Then, $W_{c}$ and $W_{s}$ are fused into a rough $W_{\mathrm{coa}}$ by a simple addition operation. $W_{coa}$ represents the Coarsely Fused Attention weights obtained by preliminarily fusing channel attention weights $W_{c}$ and spatial attention weights $W_{s}$ through simple addition operations within the CGAFusion module. After alternating $W_{\mathrm{coa}}$ and input feature X, the channels are arranged using the channel blending operation, then processed by 7×7 group convolution, and finally the σ activation function to obtain each channel to assign a unique weight as well as the final fused features. The enhanced features obtained after inputting the fused features to the MHMoE module are fed into U-Net [26] as encoder and decoder. The U-Net comprises four downsampling encoding modules and four upsampling decoding modules. Each module consists of two consecutive 3×3 convolutional layers, with each convolutional layer followed by batch normalization and a ReLU activation function. Feature maps are transmitted between the encoder and decoder through skip connections. And the output O of the MHMoE is taken as the input to obtain the final segmentation result. The teacher model network is shown in Figure 2, Module A. The loss function is shown in Equation (5).

(5) $$L_{\mathrm{Task}}^{\mathrm{teacher}}=-\sum_{i=1}^{N}t_{i}\log\left(y_{i}\right)+\sum L_{\mathrm{Dice}}\left(P_{fn},T_{f_{n}}\right).$$

where $y_{i}$ denotes the predicted probability for pixel *i* belonging to the foreground class after the network’s output layer, $P_{fn}$ represents the set of predicted probabilities for the fn-th foreground class or segmentation region, and the index fn for the second summation iterates over all the defined foreground segmentation classes for which the Dice loss ($L_{\mathrm{Dice}}$) is computed by comparing $P_{fn}$ with the corresponding ground truth $T_{fn}$.

### 2.2. Assistant Module

The teacher model uses CNN as the base module, focusing on the fine-grained extraction of local image features and precise pixel-level segmentation. However, medical images contain detailed structured semantic information. Due to the local feature extraction characteristics of CNNs, the teacher model does not fully utilize this detailed structural semantic information. Additionally, the parameters of the teacher model are frozen, making it difficult to incorporate new knowledge for self-updating. To address these two issues, this paper designs an assistant model based on the peripheral vision of the human eye (the assistant model is shown in Module B of Figure 2).

The peripheral vision mechanism of the human eye [27] refers to the ability to perceive the surrounding environment while focusing on a central object. Central vision (foveal vision) is responsible for processing details, whereas peripheral vision detects the overall environment. Peripheral vision covers the area beyond the central field of view, typically within a visual angle range of 20° to 110°. Within this range, the eye can detect movement and changes in light, although with lower visual acuity. Despite its reduced detail perception, peripheral vision is effective in recognizing object shapes and spatial layouts. The human brain integrates peripheral vision with the detailed perception of central vision to create a complete visual scene.

As far as we know, the human visual mechanism has been applied to various visual tasks, such as object detection and semantic segmentation, through models like Peripheral Vision Transformer (PVT) [28] and Perception-and-Regulation Network (PRN) [29]. However, it has not been extended to pixel-level segmentation tasks in medical imaging. Inspired by these concepts, an assistant module has been designed in this study by integrating the advantages of PVT and PRN.

Due to the uncertainty in lesion location, the input image within the assistant module’s MPA (Mimicking Peripheral Vision Attention) component is partitioned into four distinct, non-overlapping spatial regions, denoted as FA, FB, FC, and FD. This division occurs along the image’s height and width dimensions, effectively creating four quadrants: (e.g., top-left, top-right, bottom-left, and bottom-right). This simulates the saccade mechanism of the human eye, helping to detect objects that are not located at the center of the field of view. Additionally, partitioned computation significantly reduces the computational load. Thus, the assistant module not only provides complementary information but also facilitates a more dynamic and effective learning process for the student network within the collaborative framework by enabling the teacher–assistant entity to adapt. Visual feature extraction is then performed for each region, with global content features first captured using a multihead attention mechanism [30]. MHA follows the standard Transformer multihead attention mechanism proposed by Vaswani et al. [30]

(6) $$\mathrm{MSA}\left(X\right)=\underset{h\in [N_{h}]}{\mathrm{concat}}\left[{\mathrm{Self}–\mathrm{Att}}^{\left(h\right)}\left(X\right)\right]W_{\mathrm{out}}+b_{\mathrm{out}}.$$

(7) $${\mathrm{Self}–\mathrm{Att}}^{\left(h\right)}\left(X\right)=\mathrm{Normalize}\left[\Phi^{\left(h\right)}\left(X\right)\right]V^{\left(h\right)}.$$

(8) $$\Phi^{\left(h\right)}\left(X\right)=\exp\left(\tau XW_{\mathrm{qry}}^{\left(h\right)}{\left(XW_{\mathrm{key}}^{\left(h\right)}\right)}^{⊤}\right).$$

Then, the input tensor X represents the image, and the features are extracted by the convolutional kernel K. The center mask is a 3×1 matrix with a center element of 1. The weights of the convolution kernel are dynamically adjusted by the base mask $M_{\mathrm{base}}$ and the learnable mask $M_{\mathrm{learn}}$ in combination with the center mask $C_{\mathrm{mask}}$, which is a 3×3 matrix with the center element being 1 and the rest of the elements being 0.5. This mask is used to emphasize the importance of the center position of the convolution kernel to mimic the central vision of the human eye to form the adjustable mask M. This process effectively highlights the influence of the convolution kernel’s center’s influence, enabling the model to optimize the feature extraction capability during the training process. Ultimately, the tuned convolutional kernel K⊙M performs a convolutional operation with the input X to generate an output that effectively recognizes the key features of the image.

(9) $$\begin{array}{c} MPA\left(x\right)=F_{\mathrm{conv}\;}\left(x,K⊙M\right)\\ M=M_{\mathrm{base}\;}-\theta \cdot M_{\mathrm{learn}\;}\cdot C_{\mathrm{mask}\;}\cdot \left(\sum_{i=1}^{k}\sum_{j=1}^{k}K_{:,n,i\;}\right). \end{array}$$

The learnable mask $M_{learn}$ in Equation (9) is a crucial component of the Modality-Specific Perception Attention (MPA) mechanism, allowing the model to dynamically adjust the weights of the convolution kernel *K*. The $M_{learn}$ matrix is initialized with small random values drawn from a uniform distribution, specifically U(−1,1). This standard initialization practice helps in breaking symmetry and allows for effective learning from the data. The parameters within $M_{learn}$ are updated during the backpropagation process concurrently with other network parameters such as the convolution kernel *K* and the learnable scaling factor θ. The gradients for $M_{learn}$ are computed based on the overall loss function of the assistant module, $L_{Task}^{assistant}$ (as defined in Equation (11)), and an optimizer, in our case Stochastic Gradient Descent (SGD), updates its values to minimize this loss.

The MPA(x) is then normalized by multiplying it with the result of MSA(X), producing the output feature for the current region. After processing each region in the same manner, the features from different regions need to interact with each other.

(10) $$\begin{array}{c} F_{a}=C_{2}\left(\mathrm{Cats}\left(\mathrm{Parc}\left(C_{1}\left(\mathrm{Catc}\left(FA,FB,FC,FD\right)\right)\right)\right)\right)\\ F_{1}^{A}=\mathrm{softmax}\left(FA+FA*F_{a}\right). \end{array}$$

In the given context, Pars(·), Parc(·), Cats(·), and Catc(·) represent spatial dimension partitioning, channel dimension partitioning, spatial dimension concatenation, and channel dimension concatenation, respectively. C1(·) is a 3×3 convolution 1 with ReLU activation; C2(·) is a 3×3 convolution 2 with σ activation. The initial features are added, followed by a softmax layer, resulting in the interaction feature. The same process is applied to obtain the features of other regions.

The final features are encoded and decoded with CENet as the encoder and decoder and DiceLoss combined with CELoss as the loss function of the assistant model to obtain the output features and segmentation results of the assistant network. The assistant output results and the teacher network output results are weighted and added together to finally obtain the output of the teacher–assistant network for students’ online learning competition. The loss function of the assistant model is shown in Equation (11).

(11) $$L_{\mathrm{Task}}^{\mathrm{assistant}}=-\sum_{i=1}^{N}t_{i}\log\left(y_{i}\right)+\sum L_{\mathrm{Dice}}\left(P_{fn},T_{f_{n}}\right).$$

### 2.3. Competitive Knowledge Distillation

In this section, we propose a new framework that enhances the performance of student models through a dual mechanism of learning and competition. During training, the student model learns from the teacher model via knowledge distillation while continuously optimizing itself through the competition function, with the goal of surpassing the teacher model in task performance.

Step 1: Pretrain the teacher model and freeze its parameters after pretraining is completed. The pretraining process employs supervised learning using the combined loss function LTaskteacher as defined in Equation (5), which incorporates both cross-entropy loss and Dice loss components.

Step 2: Transfer knowledge from the teacher model to the student model. The knowledge distillation loss function is shown in Equation (14).

(12) $$L_{\mathrm{Task}\;}^{\mathrm{student}\;}=\frac{1}{N}\sum_{i=1}^{N}L_{\mathrm{Task}\;}\left(y_{i},{\hat{y}}_{\left(i,S\right)}\right).$$

(13) $$L_{KD}^{\mathrm{student}\;}=\frac{1}{N}\sum_{i=1}^{N}\|\Phi \left(\frac{\hat{y}\left(\cdot ,T\right)}{\tau }\right)-\Phi \left(\frac{\hat{y}\left(\cdot ,S\right)}{\tau }\right){\|}_{2}.$$

(14) $$L_{\mathrm{student}}=\alpha L_{\mathrm{Task}}^{\mathrm{student}}+\left(1-\alpha \right)L_{\mathrm{KD}}^{\mathrm{student}}.$$

Step 3: Designing Competition Functions

The goal of competitive learning is to encourage the student model to outperform the teacher model in task performance. We can design a reward function $r(x_{i})$ to achieve this goal. In this paper, we define the reward function to measure whether the student model outperforms the teacher model on the task:

(15) $$r\left(x_{i}\right)=\lambda \cdot \left(L_{\mathrm{task}\;}\left(y_{i},\hat{T}\left(x_{i}\right)\right)-L_{\mathrm{task}\;}\left(y_{i},S\left(x_{i}\right)\right)\right).$$

The competitive loss is reduced when the student model outperforms the teacher model, and the competitive loss is shown in Equation (16):

(16) $$L_{\mathrm{competitive}\;}=\sum_{i=1}^{N}\gamma_{i}\left(t\right)\cdot \delta_{i}\cdot r\left(x_{i}\right),$$

where $\gamma_{i}$ and $\sigma_{i}$ are dynamic factors that control the importance of each sample in training.

(17) $$\gamma_{i}=\frac{L_{\mathrm{task}\;}\left(y_{i},T\left(x_{i}\right)\right)}{L_{\mathrm{task}\;}\left(y_{i},T\left(x_{i}\right)\right)+L_{\mathrm{task}\;}\left(y_{i},S\left(x_{i}\right)\right)},$$

(18) $$\delta_{i}=\exp\left(\frac{L_{\mathrm{task}\;}\left(y_{i},\hat{T}\left(x_{i}\right)\right)}{\mu }\right),$$

where $\gamma_{i}$ measures the performance of the teacher model relative to the student model on a given sample. When the teacher model’s loss is low, the sample is considered easier, resulting in a relatively small value of $\gamma_{i}$. Conversely, a higher loss indicates a more difficult sample, leading to a larger value of $\gamma_{i}$. By dynamically adjusting the importance of samples, the model can focus more effectively on the more difficult samples, thus improving learning. $\delta_{i}$ is used to emphasize the impact of a particular sample. Samples with larger losses will receive higher $\delta_{i}$ values, meaning that more attention is required during training. The purpose of this is to encourage the student model to focus on those harder samples, thus helping the model to achieve improvements in overall performance. In our paper, the hyperparameters were set as λ=0.1 and μ=10.

The final loss function obtained is a combination of knowledge distillation loss, competition loss, and helper network loss, as shown in Equation (19):

(19) $$L_{\mathrm{total}}=\lambda_{\mathrm{student}}L_{\mathrm{student}}+\lambda_{\mathrm{comp}}\cdot L_{\mathrm{competitive}}+\lambda_{\mathrm{res}}\cdot L_{\mathrm{assistant}}.$$

## 3. Results

### 3.1. Materials and Methods

#### 3.1.1. Dataset

**Figure 4:**
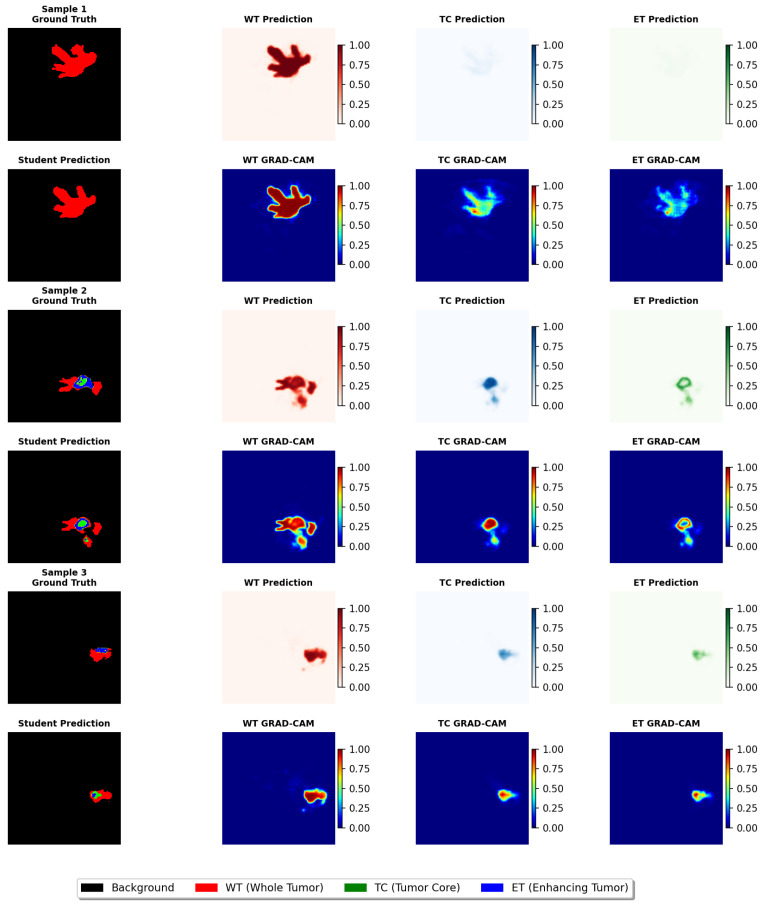
The Grad-CAM heatmap presents the GRAD-CAM visualization analysis of the TASCCNet student network for brain tumor segmentation tasks. For each sample, the first row displays, from left to right, ground truth annotations, whole tumor (WT) predictions, tumor core (TC) predictions, and enhancing tumor (ET) predictions; the second row correspondingly shows the comprehensive student network predictions and GRAD-CAM activation heatmaps for the three tumor subregions. The segmentation mask visualization adopts a standardized color encoding scheme: black represents background regions, red indicates whole tumor areas (WT), green identifies tumor core regions (TC), and blue denotes enhancing tumor areas (ET). Both prediction probability maps and GRAD-CAM heatmaps utilize normalized color scales ranging from 0 to 1, where higher values are represented by warm colors (red, yellow) and lower values by cool colors (blue).

In this paper, we utilized the BraTS 2018 [31] and BraTS 2021 datasets [32], which consist of a diverse range of brain tumor cases, covering tumors of varying types, sizes, and locations. Specifically, BraTS 2018 and BraTS 2021 include 285 and 1251 cases of multimodal MR scans, respectively, with each scan comprising four modalities: T1, T1CE, T2, and FLAIR. All scans were meticulously segmented by expert annotators and neuroradiologists, providing annotations for WT (whole tumor), ET (enhancing tumor), and TC (tumor core). Figure 4 illustrates some examples. This paper focuses on 2D segmentation, where 2D slices were extracted from the axial plane of the original data, and each slice was cropped to 160 × 160 pixels to remove unnecessary background information. The slices were then normalized to the [0, 1] range, followed by contrast and brightness enhancement. For all datasets, 80 percent of the data was used for training, 10 percent for validation, and 10 percent for testing.

#### 3.1.2. Evaluation Metrics

In the field of medical image segmentation, we employ four widely accepted evaluation metrics to comprehensively assess segmentation methods. These metrics include the Dice similarity coefficient (Dice), which is typically used to evaluate the overlap between the predicted and ground truth regions. In general, a higher Dice score indicates better segmentation performance. The Dice metric is defined as follows:

(20) $$\mathrm{Dice}=\frac{2\times TP}{2\times TP+FP+FN}.$$

where TP, FP, and FN represent the number of true positive, false positive, and false negative pixels, respectively.

#### 3.1.3. Implementation Details

In this study, we trained our TASCCNet model on a server equipped with an NVIDIA 4090D GPU (with 24 GB VRAM), a 16-core Intel Xeon (R) Platinum 8481C CPU, and 80 GB of system memory, using driver version 550.78 and CUDA 12.4. The network parameters were fine-tuned using the Stochastic Gradient Descent (SGD) optimizer. During the training phase, the initial learning rate was set to $2.0\times 10^{-4}$, and we employed the “Poly” learning rate decay strategy. The weight decay was configured as $1.0\times 10^{-4}$, with a momentum value of $9.0\times 10^{-1}$. The batch size was set to 160, and the network was trained for a total of 1000 epochs.

### 3.2. Comparison with State-of-the-Art Methods

#### 3.2.1. Quantitative Analysis

To illustrate the effectiveness of TASCCNet, we have selected state-of-the-art segmentation methods in our comparison experiments, including Multimodal Masked Autoencoders (M3AE) [17], Knowledge Distillation from multimodal to monomodal segmentation Networks (KD-Net) [19], Region-aware Fusion Network (RF-Net) [14], Heteromodal variational encoder–decoder (U-HVED) [11], Privileged Multimodal Knowledge (PMKL) [33], and Mutual Knowledge Distillation (MKD) [33,34]. Each of these modality makes a unique contribution to the field of medical segmentation.

We recognize that the performance metrics for the same method can vary across different studies due to discrepancies in data processing pipelines or specific experimental setups. Therefore, to ensure an objective and fair comparison, we first attempted to source the results directly from the original research publications. We also reimplemented the experiments according to the descriptions in those papers. If the evaluation scores from the original publication are close to our reimplemented results, we adopt the original scores. However, if there is a significant discrepancy between the original and our reimplemented results, we then use the performance scores obtained from our own reimplementation.

The teacher model performed best overall due to its access to complete multimodal inputs. It excels particularly in the whole tumor (WT) metric, achieving the highest scores across all modalities and demonstrating its Dice Score in segmenting the entire tumor. However, its performance in the enhancing tumor (ET) region was weaker, indicating limitations in segmenting the enhancing tumor. The reported results were the best achieved from multiple trials with different hyperparameters.

For the BraTS 2018 dataset, comparing our model to others highlights its clear advantages in medical image segmentation tasks. In the T1 modality, our model achieved the highest ET score (41.02), significantly outperforming models like M3AE (37.1) and RF-Net (35.3). It also showed stable performance in the TC region (63.25), close to M3AE’s best result (66.1), and ranked near the top in WT segmentation (75.53), demonstrating its well-rounded segmentation capabilities. In the T2 modality, our model continued to excel in ET segmentation with a score of 46.12, surpassing RF-Net (38.11) and KD-Net (45.66). It remained competitive in TC segmentation (66.16), approaching M3AE’s 69.4, and performed strongly in WT (83.42). In the T1ce modality, our model led in TC segmentation (78.2), surpassing M3AE (75.8) and KD-Net (78.01), and it performed nearly as well as the best in ET segmentation (71.3). Additionally, its WT performance remained consistent at 83.42. In the Flair modality, our model achieved the highest average score (64.45) among non-teacher models, excelling particularly in ET segmentation (38.9) and outperforming KD-Net (36.1) and RF-Net (36.2). It also performed strongly in WT (88.24) and TC (66.2), further demonstrating its comprehensive capability.

The “Avg” scores in Table 1 represent the mean improvement per sample in the average Dice similarity coefficient (Dice) for each modality (T1, T2, T1ce, and Flair) on the respective dataset (BraTS2018 or BraTS2021).

For the BraTS 2021 dataset, our model showed exceptional ET segmentation performance in the T1 modality, scoring 44.31 and significantly outperforming models like M3AE (40.1) and RF-Net (37.3). While slightly behind M3AE in TC segmentation (65.11 vs. 67.3), it remained competitive and outperformed others in WT segmentation (78.83). In the T2 modality, our model excelled again in ET segmentation with a score of 49.42, surpassing RF-Net (46.2) and KD-Net (46.52), showcasing its strength in capturing complex tumor regions. It also achieved a high TC score of 69.34, close to RF-Net’s 72.12, and led in WT segmentation with 86.12. In the T1ce modality, our model stood out with top scores in TC (81.14) and ET (73.3) segmentation, outperforming models like MKD (79.87) and PMKL (73). It also achieved strong WT segmentation (81.02) and an average score of 78.49, demonstrating balanced performance. In the Flair modality, our model maintained its lead, particularly in ET segmentation (41.9), far surpassing MKD (32.78) and PMKL (39.75). Additionally, it achieved the highest scores in WT (89.64) and TC (69.31) segmentation among all non-teacher models, further solidifying its dominance in this modality.

#### 3.2.2. Qualiative Analysis

**Figure 5:**
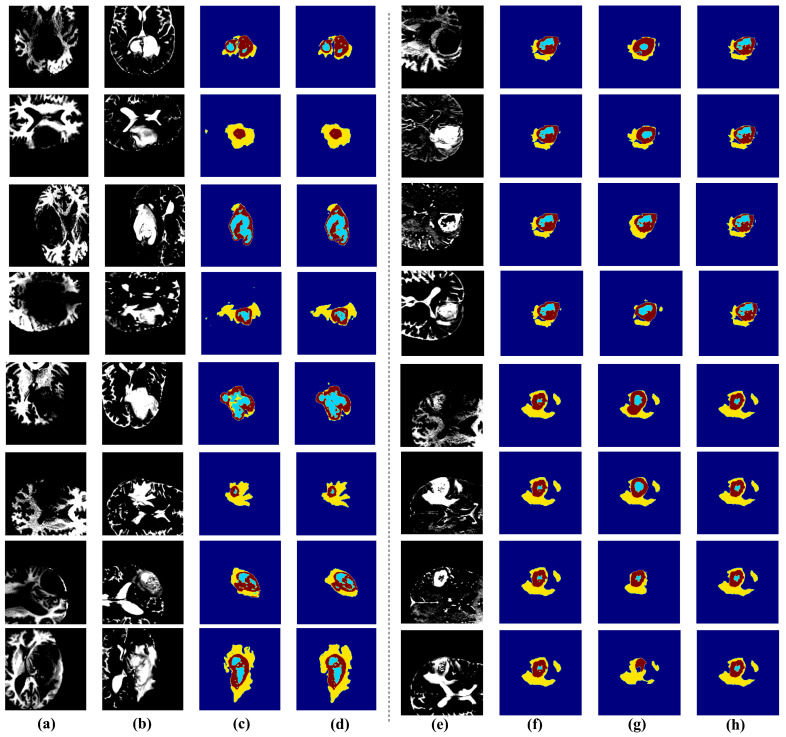
Segmentation results of teacher and student networks. (**a**–**d**) Teacher network: (**a**) T1 and T1ce inputs (channel-concatenated). (**b**) T2 and FLAIR inputs (channel-concatenated). (**c**) Teacher’s segmentation. (**d**) Ground truth. (**e**–**h**) Student network evaluation on two cases (Case 1: rows 1–4; Case 2: rows 5–8): (**e**) Student’s single-modality input (T1, Flair, T1ce, T2 per case, one modality per row). (**f**) Teacher’s segmentation for the respective case (identical across 4 rows per case). (**g**) Student’s segmentation using input from (**e**). (**h**) Ground truth for the respective case (identical across 4 rows per case).

Based on Table 1 and Table 2, we observe that the student model still has room for improvement in the T2 modality. To better understand the features and shapes, the model focuses on for different categories of images, Grad-CAM (Gradient-weighted Class Activation Mapping) heatmaps were used to highlight the regions of interest during prediction. Figure 4 presents the GRAD-CAM visualization analysis results of the student network within the TASCCNet framework conducted for model interpretability studies in brain tumor segmentation tasks. Figure 4 contains three sample cases, with each sample occupying two rows: the first row displays the ground truth annotations and prediction results for three tumor subregions, including the whole tumor (WT, red), tumor core (TC, green), and enhancing tumor (ET, blue). It should be noted that to better highlight the regions of model interest, a more vivid color mapping is employed compared to Figure 5, resulting in different color schemes between these two figures. The second row presents the student network’s comprehensive prediction results and corresponding GRAD-CAM heatmaps, where warm colors (red/yellow) indicate regions with high model attention, and cool colors (blue) represent areas with lower activation levels. Through analysis of these heatmaps, we can validate whether the model focuses on tumor-relevant anatomical structures. As shown in Figure 4, the student model effectively distinguishes the tumor from the background. However, it fails to accurately identify the peritumoral edema and enhancing tumor regions. Figure 5e–h display the segmentation results of the student network and further support this conclusion.

Figure 5 shows the segmentation results of the teacher network and student network. In the teacher network (a–d in Figure 5), the features from the four modalities—T1, T1ce, T2, and Flair—are fused and enhanced by the MHMoE, allowing the teacher model to effectively utilize the complementary characteristics of each modality. This maximizes its ability to capture various anatomical structures and pathological regions of the tumor. The integration and enhancement of these multimodal features enable the teacher model to overcome the limitations of single-modality input, achieving comprehensive tumor region segmentation with greater robustness and precision.

As shown in Figure 5e–h, compared to the teacher network, the student network lacked complementary information from multiple modalities and cannot capture sufficient comprehensive data, resulting in weaker performance when segmenting certain key regions. However, thanks to the structural information provided by the assistant network, the student model still performed quite well, even in situations where only a single modality was available.

When using the T1 modality, the student model performed well in segmenting the whole tumor (WT) and tumor core (TC) regions but struggled with the enhancing tumor (ET) region, leading to some missegmentation. With the Flair modality, the model effectively segmented the edema region, but its ability to detect the tumor core was limited, and it failed to accurately segment the enhancing tumor. When provided with T1ce input, the model’s Dice Score in segmenting the enhancing tumor (ET) improved, but it still showed weaknesses in the tumor core and whole tumor regions. With T2 modality input, the student model performed relatively well in segmenting the whole tumor (WT), but its performance in identifying the tumor core and enhancing tumor (ET) remained suboptimal, particularly in capturing finer details.

### 3.3. Ablation Experiments

#### 3.3.1. MHMoE

As shown in Table 3, when only the MHMoE module was activated (first row), significant improvements in the Dice scores could be observed. On the BraTS2018 dataset, the T1 score was 1.24, T2 was 1.1, T1ce was 1.2, and Flair was 0.98. Similarly, on the BraTS2021 dataset, the T1 score was 2.41, T2 was 1.07, T1ce was 0.54, and Flair was 1.23. These results demonstrate that the MHMoE module significantly enhances the model’s performance in segmenting different tumor regions by effectively integrating multimodal features, especially in the T1 and T2 modalities. Moreover, when combined with other components such as the assistant network or the competitive mechanism, MHMoE’s performance was further boosted. For instance, when MHMoE was combined with the competitive mechanism, the average score for the Flair modality on the BraTS2021 dataset increased from 0.98 to 1.204. This shows that MHMoE, when used alongside other mechanisms, can effectively improve the model’s ability to recognize complex modalities.

#### 3.3.2. Assistant

When the assistant network was added alone, the average Dice scores for the T1 modality in the BraTS2018 and BraTS2021 datasets increased by 2.12 and 2.62, respectively, with Flair scores rising by 1.74 and 2.82. This shows the assistant network enhances the model’s ability to capture structural information, even when single-modality data are missing. Combining it with other modules further boosted performance. For instance, pairing the assistant network with MHMoE increased the T2 score in the BraTS2021 dataset from 1.07 to 1.98, demonstrating its effectiveness in handling complex modality information, especially in T2 and Flair modalities. Without MHMoE, the assistant network and the competitive mechanism complement each other to improve single-modality segmentation, but their ability to handle multimodal integration remains limited. This underscores the assistant’s critical role in the collaborative learning process, especially for scenarios with missing modalities.

#### 3.3.3. Compete

When the competitive mechanism was applied alone, improvements in the T1 modality were modest, with increases of 0.3 and 1.12 in the BraTS2018 and BraTS2021 datasets, respectively, showing its limited impact on its own. However, when combined with the assistant network or MHMoE, the performance improved significantly. For example, pairing the competitive mechanism with the assistant network increased the Flair score in the BraTS2021 dataset from 1.74 to 1.79, indicating that it helps the student model focus more effectively on difficult-to-segment areas.

The experimental results clearly show that MHMoE, the assistant network, and the competitive mechanism each contribute to segmentation performance improvements. When combined, they further enhance overall model performance, particularly in recognizing complex tumor regions, leading to greater robustness and Dice Score across different modalities.

### 3.4. Discussion

Based on the qualitative and quantitative results, two areas for improvement have been identified in this study. The first is the design of the competitive function. It is intuitive that competition can enhance model performance, as seen in the “adversarial” mechanism of GANs, which has achieved significant success in visual tasks. Although the competitive function in this study can combine with other modules to improve the segmentation Dice score, the improvement is relatively limited. Therefore, further optimization of the loss function based on the idea of competition is necessary.

The second area concerns the issue of class imbalance, particularly for small-lesion segmentation. In medical imaging, lesions are often small, which causes the model to focus more on the background, resulting in class imbalance. Additionally, artifacts and noise are commonly present in actual medical images due to imaging equipment and other factors, further complicating the segmentation process. The challenge of accurately segmenting smaller and less prevalent tumor subregions, such as the enhancing tumor (ET), is also reflected in our findings (Table 1 and Table 2), where the Dice scores for ET were consistently lower than those for WT and TC across most methods. This observation underscores the impact of inherent class imbalance and the difficulty in delineating fine-grained details of small lesions, a persistent issue in medical image segmentation. This difficulty is particularly pronounced in a 2D slice-by-slice approach, which may fail to capture the 3D continuity of small, disparate enhancing regions. A future transition to a 3D model architecture is a promising direction to better leverage volumetric context and mitigate this issue. Improving the model to address small lesion segmentation remains a challenge that we aim to solve in future work.

Furthermore, a detailed sensitivity analysis of the hyperparameters λ and μ within the competitive loss function could provide deeper insights into their optimal settings and impact on training dynamics, which remains an avenue for future investigation. While this study focused on segmentation accuracy, we acknowledge that a comprehensive analysis of computational costs, including FLOPs and inference time, is a critical next step for assessing clinical viability. We plan to conduct this analysis in subsequent research.

Dataset limitations must be considered, as BraTS 2018 and BraTS 2021, while established benchmarks, may have constrained institutional and geographical representation. Scanner variations in models, manufacturers, and acquisition parameters present universal medical imaging challenges that can cause domain shift effects. Validation across additional datasets is necessary to establish broader model generalizability.

## 4. Conclusions

In this study, the TASCCNet model is proposed, consisting of an updated teacher–assistant network and a student network competing against the teacher network. The network is designed when only a single modality is available. MHMoE is introduced to enhance feature fusion. For knowledge updating, an assistant model based on the principles of human visual perception is designed. The student model learns from the teacher network while simultaneously competing with it. Experiments on the BraTS2018 and BraTS2021 datasets demonstrate the stability and accuracy of our model. Finally, the strengths and weaknesses of the model have been evaluated.

While Zhang et al. [35] explored mixture of modality experts for brain lesion segmentation with similar conceptual foundations, our work introduces distinct contributions through the Multihead Mixture of Experts (MHMOE) module, which incorporates a multihead mechanism for finer-grained parallel processing of channel feature subsets and employs convolutional expert networks that preserve spatial image structure.

In future research, to ensure the reproducibility of experiments, we plan to conduct multiple repeated experiments in the future to obtain the error range of the results. And Flops calculation is necessary. We plan to extend the proposed network in future works and handle three-dimensional (3D) images, improving its ability to capture spatial relationships and enhancing the accuracy of segmentation and detection tasks. Additionally, we envision applying the model to other challenging domains, such as natural image segmentation and object detection.

## Figures and Tables

**Table 1:** Comparison of segmentation performance (Dice Score, %) on the BraTS 2018 dataset for single-modality inputs. This table presents a performance comparison of different methods on the BraTS 2018 dataset when utilizing only one of the four single-MRI modalities. The table compares models, including the baseline “Teacher” model (using full multimodal input) and our proposed “Our” model (using single-modality input). Performance is evaluated using the Dice (presented as a percentage, %), with scores provided separately for the different MRI modalities, along with an average (Avg) of these three scores. WT stands for whole tumor, TC for tumor core, and ET for enhancing tumor. Higher values indicate better segmentation performance.

Method	T1				T2				T1ce				Flair			
	WT	TC	ET	Avg	WT	TC	ET	Avg	WT	TC	ET	Avg	WT	TC	ET	Avg
Teacher	89.77	82.93	70.65	81.12	–	–	–	–	–	–	–	–	–	–	–	–
M3AE [17]	74.4	**66.1**	37.1	59.20	84.8	**69.4**	47.6	67.27	78.01	80.13	66.3	74.81	88.1	64.4	36.1	62.87
KD-Net [19]	72.62	59.13	38.1	56.62	82.47	66.17	45.66	64.77	73.6	**81.5**	67.7	74.27	85.8	62.6	38.1	62.17
RF-Net [14]	74.8	65.2	35.3	58.43	**84.9**	66.1	38.11	63.04	67.8	73.21	67.2	69.40	77.3	51.1	20.8	49.73
U-HVED [31]	54.4	41.7	12.3	36.13	81.8	57.66	38.7	59.39	67.8	73.21	67.2	69.40	77.3	51.1	20.8	49.73
PMKL [32]	71.31	65.2	38.37	58.29	82.1	67.82	48.1	66.01	70.5	75.92	70.36	72.23	84.64	64.56	36.35	61.85
MKD [33]	74.71	59.49	39.31	57.84	81.43	66.7	45.5	64.54	72.96	77.41	70.82	73.75	86.8	63.77	34.5	61.69
Our	**75.53**	63.25	**41.02**	**59.93**	83.42	66.16	46.12	65.23	**78.2**	80.14	**71.3**	**76.55**	**88.24**	**66.2**	**38.9**	**64.45**

**Table 2:** Comparison of segmentation performance (Dice Score, %) on the BraTS 2021 dataset for single-modality inputs. This table presents a performance comparison of different methods on the BraTS 2021 dataset when utilizing only one of the four single-MRI modalities. The table compares models, including the baseline “Teacher” model (using full multimodal input) and our proposed “Our” model (using single-modality input). Performance is evaluated using the Dice (presented as a percentage, %), with scores provided separately for the different MRI modalities, along with an average (Avg) of these three scores. WT stands for whole tumor, TC for tumor core, and ET for enhancing tumor. Higher values indicate better segmentation performance.

Method	T1				T2				T1ce				Flair			
	WT	TC	ET	Avg	WT	TC	ET	Avg	WT	TC	ET	Avg	WT	TC	ET	Avg
Teacher	90.67	84.37	72.1	82.38	–	–	–	–	–	–	–	–	–	–	–	–
M3AE [17]	77.82	**67.3**	40.1	61.74	85.8	71.25	48.61	68.55	77.84	81.9	72.43	77.39	86.8	68.2	40.29	65.10
KD-Net [19]	75.82	62.18	42.74	60.25	84.8	67.57	46.52	66.30	79.86	**82.73**	68.32	76.97	87.1	62.82	38.92	62.95
RF-Net [14]	77.1	66	37.3	60.13	**86**	**72.12**	46.2	68.11	76.7	82.5	68.2	75.80	87.3	69.1	38.1	64.83
U-HVED [31]	55.13	43.81	19.95	39.63	80.02	62.35	37.3	59.89	68.5	73	66.6	69.37	81.91	52.92	21.32	52.05
PMKL [32]	74.32	66.7	41.22	60.75	84.1	70.32	48.83	67.75	74.71	79.87	72.57	75.72	85.86	67.47	39.75	64.36
MKD [33]	76.47	63.12	43.15	60.91	83.43	69.43	45.9	66.25	74.62	80.12	71.9	75.55	86.1	67.1	32.78	61.99
Our	**78.83**	65.11	**44.31**	**62.75**	86.12	69.34	**49.42**	**68.29**	**81.02**	81.14	**73.3**	**78.49**	**89.64**	**69.31**	**41.9**	**66.95**

**Table 3:** Ablation study of TASCCNet components: Average Dice score improvement (%) on BraTS 2018 and BraTS 2021 datasets for single-modality inputs. This table presents the results of an ablation study conducted on key components of our proposed TASCCNet framework: specifically, the MHMOE module, the assistant network, and the competitive learning mechanism. The table illustrates the impact on the student model’s average Dice scores when these components are systematically removed from or retained within the overall training and knowledge transfer process, evaluated on both the BraTS 2018 and BraTS 2021 datasets using single-modality inputs. The values in the table represent the improvement in the student model’s average Dice score in percentage points (p.p.) for different systemic configurations compared to a baseline scenario, where the student model was trained without the benefits of these specific TASCCNet components.

MHMoE	Assistant	Compete	BraTS2018				BraTS2021			
			T1 Avg	T2 Avg	T1ce Avg	Flair Avg	T1 Avg	T2 Avg	T1ce Avg	Flair Avg
🗸	×	×	1.24	1.10	1.20	0.98	2.41	1.07	0.54	1.23
×	🗸	×	2.12	1.98	1.43	1.74	2.62	1.18	0.42	2.82
×	×	🗸	0.30	0.96	0.26	0.75	1.12	−0.45	0.26	0.75
🗸	🗸	×	2.76	2.34	1.78	1.92	2.74	1.98	1.66	3.14
🗸	×	🗸	2.35	2.17	1.33	1.20	2.58	1.55	1.33	1.98
×	🗸	🗸	2.11	1.97	1.20	1.57	2.67	1.61	0.75	1.79
🗸	🗸	🗸	2.81	3.01	1.92	1.89	3.13	2.05	1.74	3.61

## Data Availability

The BraTS 2021 brain tumor segmentation dataset was obtained from the RSNA-ASNR-MICCAI Brain Tumor Segmentation (BraTS) Challenge 2021, which is publicly available at https://www.kaggle.com/datasets/dschettler8845/brats-2021-task1 (accessed on 15 September 2024). The BraTS18 brain tumor segmentation dataset was derived from the Multimodal Brain Tumor Segmentation Challenge 2018 and can be accessed at https://www.kaggle.com/competitions/rsna-miccai-brain-tumor-radiogenomic-classification (accessed on 15 September 2024).
